# Frequency of Guideline-Discordant Prostate Cancer Screening Among Older Males

**DOI:** 10.1001/jamanetworkopen.2024.8487

**Published:** 2024-04-25

**Authors:** Kevin H. Kensler, Jialin Mao, Meenakshi Davuluri

**Affiliations:** 1 Department of Population Health Sciences, Weill Cornell Medicine, New York, New York; 2 Sandra and Edward Meyer Cancer Center, Weill Cornell Medicine, New York, New York; 3 Department of Urology, Weill Cornell Medicine, New York, New York

## Abstract

This cross-sectional study examines the association of life expectancy and prostate cancer screening practices among older males using data from a national survey.

## Introduction

Guidelines currently recommend shared decision-making between males and their health care clinicians regarding prostate cancer screening using prostate-specific antigen (PSA), taking into consideration patient age and life expectancy.^1^ However, it is unclear the extent to which life expectancy is associated with screening practices among older males. We estimated the frequency of PSA-based screening by patient age and estimated life expectancy using data from a national survey.

## Methods

This cross-sectional study followed the Strengthening the Reporting of Observational Studies in Epidemiology (STROBE) reporting guideline. Informed consent was waived because publicly available deidentified participant data were used. The study was deemed exempt from review by the Weill Cornell Medicine institutional review board.

The proportion of males who self-reported undergoing a screening PSA test in the prior 2 years was estimated among males ages 60 years or older without a history of prostate cancer in the 2020 Behavioral Risk Factor Surveillance System (BRFSS), which is a population-based survey administered by the US Centers for Disease Control and Prevention that is generalizable to the community-dwelling US adult population.^2^ The 2020 BRFSS is the most recent BRFSS to ask about prostate cancer screening in a core module.

A modified version of the 10-year mortality index was implemented.^3,4^ Estimated mortality risk scores (potential range of 3.5 to 24.5 in this population) were categorized as 5.5 or less, 6.0 to 7.5, 8.0 to 9.5, or 10.0 or greater, corresponding to the estimated 10-year mortality of less than 30%, 30% to 51%, 52% to 70%, and 71% or more, respectively. Accounting for the complex BRFSS survey design, we estimated the proportion of males who received a PSA test for screening purposes in the prior 2 years by respondent age and estimated mortality risk. Multivariable logistic regression models were fit to estimate odds ratios (ORs) and 95% CIs of receiving PSA-based screening by age and estimated mortality risk, adjusting for factors influencing health care access and use, including race and ethnicity, health insurance status, annual income, educational attainment, and marital status. All hypothesis tests were 2-sided with *P* < .05 considered statistically significant. All analyses were conducted in SAS version 9.4 (SAS Institute).

## Results

Of 401 958 BRFSS respondents, 57 397 met eligibility criteria, of whom a weighted 31.7% were aged 60 to 64 years, 23.8% were aged 65 to 69 years, 19.2% were aged 70 to 74 years, 13.0% were aged 75 to 79 years, and 12.3% were aged 80 years or older. The weighted percentage of males with a mortality risk score of 5.5 or less was 35.7%, a score of 6.0 to 7.5 was 25.4%, a score of 8.0 to 9.5 was 19.6%, and a score of 10 or more was 19.4%. The estimated 2-year prostate cancer screening rates were 36.3% (95% CI, 34.5%-38.2%) among participants aged 60 to 64 years, 42.8% (95% CI, 40.8%-44.8%) among those aged 65 to 69 years, 47.1% (95% CI, 44.9%-49.2%) among those aged 70 to 74 years, 42.7% (95% CI, 39.6%-45.9%) among those aged 75 to 79 years, and 30.4% (95% CI, 27.9%-32.8%) among those aged 80 years or older (Figure). Screening frequencies decreased from 43.4% (95% CI, 41.6%-45.2%) among males with the greatest life expectancy to 30.4% (95% CI, 28.2%-32.7%) among those with the lowest life expectancy (Figure). Within age groups, the frequency of prostate cancer screening decreased with lower life expectancy (Figure and Table). The odds of undergoing PSA-based screening were 20% to 43% lower comparing categories with the lowest life expectancy with those with the highest life expectancy across age groups. However, the estimated screening frequencies among males in the category with the lowest estimated life expectancy, corresponding to an estimated 71% or greater risk of death within 10 years, were still greater than 20% in all age groups.

**Figure.**
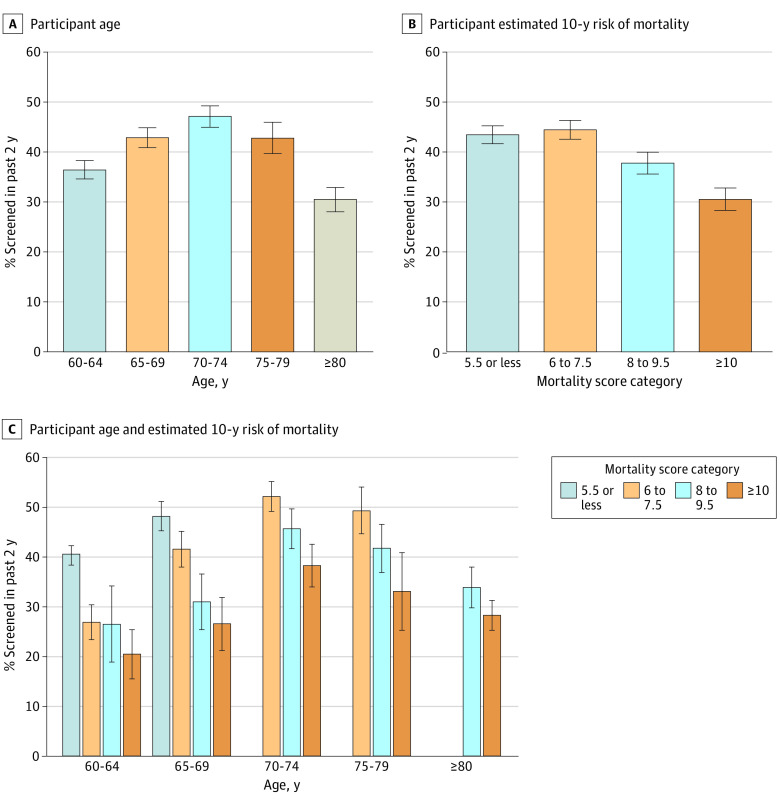
Proportion of Males Aged 60 Years or Older Without a History of Prostate Cancer Who Self-Reported Receiving a Screening Prostate-Specific Antigen Test in the Past 2 Years in the 2020 Behavioral Risk Factor Surveillance System The minimum possible mortality scores are 6.5 and 8.5 for males ages 70 to 79 years and 80 years older, respectively. Error bars indicate 95% CIs.

**Table.** Odds of Receipt a Screening PSA Test Within the Past 2 Years by Age and Estimated 10-Year Mortality Risk in the 2020 BRFSS

Participant age, mortality score category and estimated 10-y mortality^a^	OR (95% CI)^b^
60-64, y	
5.5 or Less and less than 30%	1 [Reference]
6 to 7.5 and 30% to 51%	0.71 (0.57-0.88)
8 to 9.5 and 52% to 70%	0.75 (0.48-1.17)
10 or Greater and 71% or greater	0.68 (0.46-0.99)
65-69, y	
5.5 or Less and less than 30%	1 [Reference]
6 to 7.5 and 30% to 51%	0.89 (0.75-1.05)
8 to 9.5 and 52% to 70%	0.71 (0.55-0.92)
10 or Greater and 71% or greater	0.66 (0.48-0.90)
70-74, y	
5.5 or Less and less than 30%^c^	NA
6 to 7.5 and 30% to 51%	1 [Reference]
8 to 9.5 and 52% to 70%	0.81 (0.67-0.99)
10 or Greater and 71% or greater	0.74 (0.59-0.93)
75-79, y	
5.5 or Less and less than 30%^c^	NA
6 to 7.5 and 30% to 51%	1 [Reference]
8 to 9.5 and 52% to 70%	0.75 (0.57-0.98)
10 or Greater and 71% or greater	0.57 (0.42-0.78)
≥80, y	
5.5 or Less and less than 30%^c^	NA
6 to 7.5 and 30% to 51%^c^	NA
8 to 9.5 and 52% to 70%	1 [Reference]
10 or Greater and 71% or greater	0.80 (0.64-1.01)

Abbreviations: BRFSS, Behavioral Risk Factor Surveillance System; NA, not available; OR, odds ratio; PSA, prostate-specific antigen.

^a^ Estimated 10-year mortality is based on the index developed by Cruz et al^3^ and adapted by Moss et al^4^ for the BRFSS.

^b^ OR is adjusted for race and ethnicity, health insurance status, annual income, educational attainment, employment status, marital status.

^c^ The minimum possible mortality scores are 6.5 and 8.5 for males ages 70 to 79 years and 80 years or older, respectively.

## Discussion

Our findings indicate that many males aged 70 years and older or those with a high risk of death within 10 years undergo prostate cancer screening despite the recommendation against screening in these populations by current guidelines. Given the long natural history of prostate cancer and lead time associated with PSA-based screening, these males have a low likelihood of receiving any mortality benefit from continued screening yet face the potential harms of overdiagnosis, such as complications after prostate biopsy for a false-positive screening and psychological stress associated with a cancer diagnosis.^5^ PSA-based screening rates have increased among males aged 70 years and older in recent years, suggesting that enhancements to the shared decision-making process are needed to ensure that older males who undergo screening are those who may potentially benefit.^6^ Limitations of this work include the reliance on self-reported screening history and potential measurement error in estimating life expectancy.

## Notes

Supplement.
Data Sharing Statement
