# Supplementary material for: Frequency of Guideline-Discordant Prostate Cancer Screening Among Older Males
Source: JAMA Netw Open. 2024 Apr 25;7(4):e248487. doi: 10.1001/jamanetworkopen.2024.8487 (PMC11046335; doi:10.1001/jamanetworkopen.2024.8487)
Supplement: Supplement. — Data Sharing Statement [file jamanetwopen-e248487-s001.pdf]

Data Sharing Statement

Data
Data available: No

Additional Information
Explanation for why data not available: Data from the BRFSS are publicly available from the CDC.
